# Precision Prevention: Using Data to Target the Right Intervention at the Right Intensity in the Right Community at the Right Time

**DOI:** 10.1055/s-0044-1800713

**Published:** 2025-04-08

**Authors:** Evelyn Gallego, Eugenia McPeek Hinz, Bria Massey, Elizabeth Cuervo Tilson, Jessica D. Tenenbaum

**Affiliations:** 1 EMI Advisors; 2 Duke University Health System; 3 John Hopkins University; 4 North Carolina Department of Health and Human Services; 5 North Carolina Department of Health and Human Services, Duke University

**Keywords:** Public Health, Preventive Health, Social Determinants of Health, Population Health

## Abstract

**Objectives** : This survey paper summarizes the recent trend of “Precision Prevention” in public health, focusing on significant developments in informatics to enable targeted prevention and improved public health.

**Methods** : Given relatively limited use of the term “Precision Prevention” in the literature to date, com-bined with significant developments in this space outside of peer reviewed literature, the topic was ill-suited for a systematic review approach. Instead, the co-authors used a narrative review approach, combining related search terms and complementary expertise to develop and refine sub-topics to be included. Each section was then written using a combination of prior knowledge and specific relevant search terms.

**Results** : The paper opens with an explanation of the term “precision prevention”, including its origins and relationship to other concepts such as precision medicine. It then provides an overview of types of data relevant to precision prevention, as well as how those data are collected in different contexts and through different modalities. The authors then describe the HL7 Gravity Project, a multi-stakeholder public collaborative project aimed at data standardization in the social determinants space. Finally, the authors present how those data types are used across the spectrum from clinical care to target outreach for human services, to data-driven health policy.

**Conclusions** : Precision prevention, targeting the right intervention to the right population at the right time, is now recognized as of vital importance, particularly in light of the COVID-19 pandemic's spotlight on health disparities and societal consequences. Optimizing interventions targeted at different communities and populations will require novel and innovative collection, use, and dissemination of data, information, and knowledge. The talent and skills of the international informatics community are critical for success in this work.

## 1. Introduction

In the years following the complete sequencing of the human genome, much attention was given to the idea of personalized medicine and related efforts— genomic medicine, P4 medicine (predictive, preventive, personalized, and participatory), and later, *precision medicine* [ 1 2 3 4 ]. The latter term was favored following the announcement in President Obama's 2015 State of the Union address of a new federal “Precision Medicine Initiative,” since rebranded as the “All of Us” project [ 4 , 5 ]. Even from the early days of the precision medicine era, despite the frequently used picture of President Obama next to a molecular model of DNA [ 6 ], it was recognized that DNA is only a small part of the picture— environment and lifestyle are critical components as well [ 7 ].

To understand what is meant by “precision prevention”, it may be useful to examine this term in the context of related terms, as well as some axes that they connote. Key related terms include *precision medicine, precision population health* , and *precision public health* , as well as *public health* and *population health* without the qualifier. It should be noted that these terms are not necessarily used consistently, nor does everyone agree on specific definitions or nuanced differences. However, different combinations of these terms may connote certain nuances including target audience (individuals, groups, or entire populations), level of well-being (from ill to thriving), type of intervention (primary prevention *vs.* secondary or tertiary prevention *vs.* treatment), level of risk (from population average to someone experiencing an adverse event or outcome), and responsible actor ( *e.g.* , clinical care provider *vs.* governmental policy makers) (see Box 1).

### 1.1 Public Health vs. Population Health

The National Academy of Medicine has defined public health as “ *what we as a society do collectively to assure the conditions in which people can be healthy* ” [ 8 ]. The CDC has defined population health, in contrast, as “ *an opportunity for health care systems, agencies and organizations to work together in order to improve the health outcomes of the communities they serve* ” [ 9 ]. The distinction was summed up by the Massachusetts Public Health Commissioner, Dr. Monica Bharel, in a 2020 interview by saying, “ *Public health is about what we're doing as a society, and population health is about what a system is doing for their community* ” [ 10 ]. However, Dr. Bharel goes on to point out that the distinction is largely artificial and, in fact, does a disservice to patients and communities by perpetuating siloed services and solutions [ 10 ]. In either case, the focus is on groups of people— whether a specific population or an entire society—and while the work spans prevention to treatment (healthcare systems are often not involved until there is an issue to be treated), there is an emphasis on prevention, particularly in the context of value-based care. Beyond simply prevention of disease, both definitions incorporate aspects of health promotion. With the aspiration that people “be healthy” and have improved “health outcomes”, public and population health aim not only to promote the absence of illness but an enhanced state of well-being [ 11 ].

### 1.2 Precisely what do we mean by Precision?

The addition of the “precision” modifier tends to connote two additional aspects: first, the use of more and more varied types of data to inform the practice, whether medicine, public health, or population health.[ 12 , 13 ] And second, the targeting of the practice to a more specific audience, whether an individual or a group.[ 4 , 13 ] Precision *medicine* shares with *personalized medicine, stratified medicine* , and even *genomic medicine* the use of greater amounts of data (clinical, physiologic, genomic, lifestyle, and environment) to guide the right intervention for the right person (or group of people) at the right time. In the same way, precision *prevention* (and precision public and population health) attempts to use data to refine traditional prevention and health promotion approaches to better target specific groups within a community. *Precision* can refer to an individual, but it may also refer to groups of people. At the extreme, it can be thought of as anything short of the entire population. The terms “precision medicine” and “personalized medicine” were meant to be juxtaposed with the term “one size fits all” medicine, suggesting that medical treatment must be tailored to the individual and not merely following a single guideline. Of course, clinicians will rightfully point out that medicine has always been personalized in practice.[ 14 ]

### 1.3 All together now: defining Precision Prevention

The term *precision prevention* was first coined in the context of cancer and defined as involving the “ *use of biological, behavioral, socioeconomic, and epidemiologic data* ” [ 15 ] to reduce cancer incidence and mortality. Later, a distinction was made between precision *treatment* and precision *prevention* , with the latter applied “ *‘above the skin’ to overcome psychosocial barriers, emphasize achievable goals, or adapt to families' differing economic or cultural circumstances* ” [ 16 ]. Without actually using the term “precision prevention”, Pearson *et al.* explore all relevant issues, and pose a vision for precision public health as “ *the right intervention at the right intensity in the right community at the right time* ” [ 17 ]. We therefore propose the following definition for precision prevention in the context of this survey paper: “the use of biologic, behavioral, socioeconomic, and epidemiologic data to inform the right intervention at the right intensity in the right community at the right time, in order to prevent or reduce illness and improve health”.

**Figure 1:**
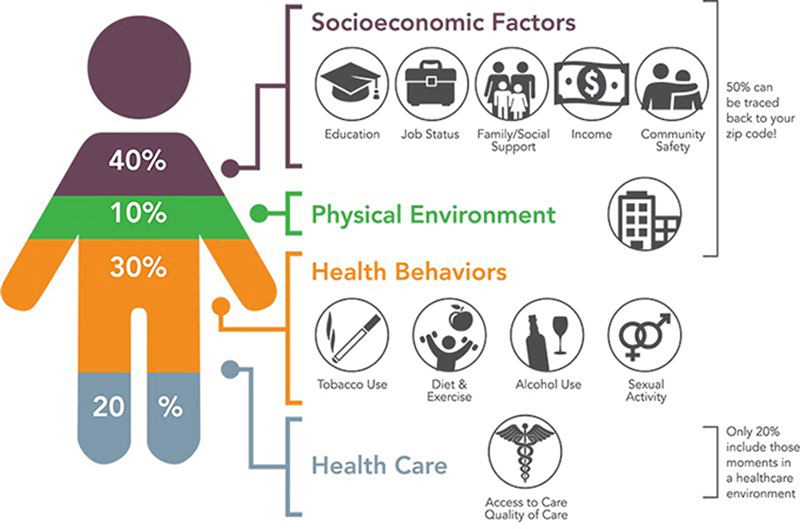
Relative contribution of socioeconomic factors, physical environment, health behaviors, and health care toward the health of an individual. (Figure source: https://coveragetoolkit.org/health-equity-and-the-national-dpp/defining-health-equity ). Prepared under Cooperative Agreement Number 5NU38OT000225-04, funded by the Centers for Disease Control and Prevention).

As illustrated in Figure 1 , it is estimated that 80% of health is driven by a combination of social and economic factors ( *e.g.* , income and employment), health behaviors ( *e.g.* , diet and exercise), and physical environment ( *e.g.* , housing and transportation) [ 18 , 19 ]. Consequently, access to high quality clinical care is necessary but not sufficient for good health. The combination of non-clinical drivers of health are often referred to as social determinants of health (SDOH) in the context of a group or community and can have both positive and negative influences on health. In contrast, health related social needs (HRSNs) are more specific when referring to a specific family or an individual [ 20 , 21 ]. To ensure every individual has the opportunity for health, it is essential to tackle *all* of the influencing factors.

**Figure 2:**
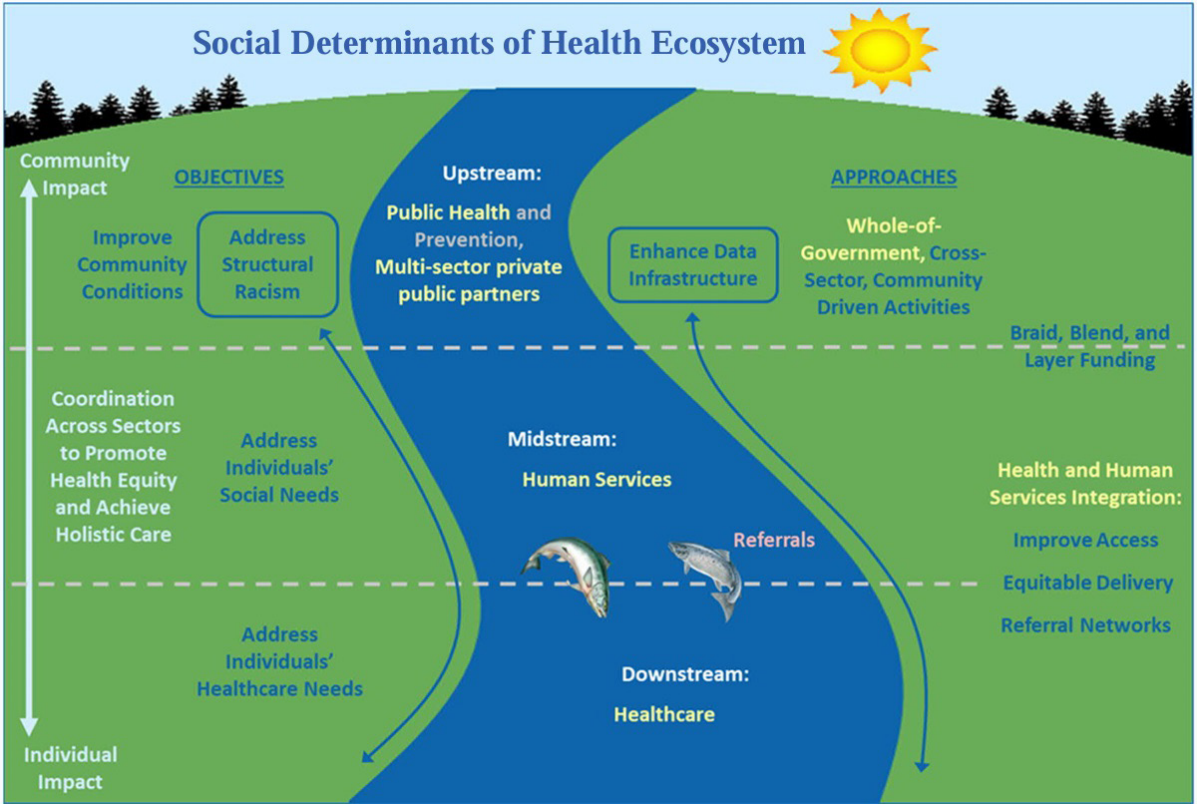
The Social Determinants of Health (SDOH) Ecosystem, as depicted in [ 22 ].

In November 2023, the U.S. Office of Science and Technology Policy released a “Playbook” to address SDOH and HRSNs [ 20 ]. This report describes an “ecosystem” of factors that impact health, as depicted in Figure 2 . The bottom third of the figure represents individuals and their healthcare. The top two thirds of the figure— community conditions, environment, and individuals' social needs— are the primary domain of both precision prevention and precision health promotion.

Note that while social needs were not previously considered “targetable” for prevention, recent developments in SDOH referral (and reimbursement) are in fact enabling intervention in these areas, e.g., housing, transportation to help prevent (or mitigate) disease. Additionally, community conditions and environment, while not generally actionable *per se* , can help identify targeted populations and communities for outreach and services.

The precision prevention framework, and prevention in general, can be applied using a tiered risk model: 1) the full population, 2) higher risk or “vulnerable” populations, and 3) populations already experiencing adverse outcomes [ 23 , 24 ]. In this way, precision prevention may be prophylactic, aimed at preventing disease in the first place, or mitigating, aimed at reducing the severity or frequency of adverse events for afflicted individuals. Both across population risk tiers, and through the upstream, midstream, and downstream domains depicted in Figure 2 , novel and innovative collection, exchange, and use of data is required.

For this reason, it is not surprising that the first of three “pillars” of action recommended in the SDOH Playbook [ 20 ] is to “ *Expand Data Gathering and Sharing* .” Precision prevention requires innovation of data infrastructure and capabilities at all levels. First, data relating to SDOH and social needs must be defined. Once the appropriate data elements are identified, they must be collected in a standardized way. Finally, data analytics and decision support can be applied across tiers to inform interventions tailored to individuals and populations. The sections below delve into each of these three areas.

### 1.4 A few Words on Scope

Note that the definition above includes biologic and epidemiological data. However, full coverage of all of these data types would require far more space than this survey permits. Fortunately, the collection and use of biologic and epidemiological data are already well covered in the precision medicine and public health literature. In this survey, we emphasize behavioral and socioeconomic data as they are the novel aspects of the combined concepts of *precision* and *prevention* .

It should also be noted that while data are a primary focus in the field of informatics, they represent just one aspect of current barriers to the realization of precision prevention. Policy issues, misaligned financial incentives, evidence gaps, skills gaps, siloed actors, and subconscious bias all represent considerable obstacles to progress in this space. However, in the context of the International Medical Informatics Association's *Yearbook of Medical Informatics* , we focus on data-related challenges and achievements.

## 2. Methods

The concept of “Precision Prevention” in the context of public health is novel enough that a search for (“precision prevention” AND “public health”) in PubMed in the final days of 2023 returns only ~100 hits, only 19 of which were published in 2023, 26 in 2022. Many of those articles focused on topics that fall under the more commonly used term “precision medicine.” Conversely, many relevant publications do not actually use the term “precision prevention.” In addition, many of the notable data-related developments in this space over the past 1-2 years have been outside of peer reviewed literature, including federal regulations, funded initiatives, and health information technology (IT) trends. This emerging topic is thus better suited for a narrative review approach than for a systematic review.

The authors first performed searches in PubMed and Google Scholar on variations of related terms, *e.g.* , “precision population public health”, “(data OR informatics) AND SDOH”, “precision public health data”. An outline was developed using a combination of themes from the resulting articles and the authors' perspectives and expertise. Sections were assigned based on respective expertise. The Gravity section was written based entirely on author (EG) expertise. Each other section was written using a combination of prior knowledge and relevant search terms for that portion of the literature, *e.g.* , “SDOH AND mHealth”, “SDOH AND EHR”. The sections were then combined and edited for cohesion and space limitations.

## 3. Social Needs Data

We cannot improve what we do not measure. Just as there are several factors that can affect a person's health risks, there are several data domains that become relevant for a precision prevention approach. For precision prevention, we first need to consider what data is important to gather at the individual level that incorporates social needs, preferences, and person-centered goals [ 25 ].

Geospatial information can give clues to a person's relative economic resources, proximity to health providers or grocery stores, or exposure to pollution or contaminants. Demographic data, including age, race, ethnicity, language, sexual orientation, and gender identity, can provide context for a person's cultural experience, or societal biases they may encounter on a regular basis. Socioeconomic data, along with insurance status, similarly give clues regarding multiple risk factors, including financial strain and access to care. And disability status can illuminate other ways in which a person might be part of a community with additional challenges. Some of these elements ( *e.g.* , race, ethnicity, preferred language, sex assigned at birth) were included as demographic data elements in the first version of the U.S. Core Data for Interoperability (USCDI— see below for more information) [ 26 ]. Others, such as gender identity, sexual orientation, and socioeconomic factors were only added in USCDI Version 2. Disability status was added in Version 3, but some have noted concern with the fact that it falls under health status rather than demographics. They also note a lack of standards to use for this data element [ 27 ].

In addition to the types above, the past decade or so has seen the collection and use of SDOH-specific data types: assessments, problems, goals, and interventions. SDOH assessment data may be collected explicitly through a health questionnaire, coded using LOINC, or collected implicitly through patient encounter notes. SDOH intervention data might take the form of referrals for services documented in an electronic health record (EHR) or related clinical IT system. Referrals may be thought of as a “prescription” for services to address a social need, *e.g.* , housing instability or food insecurity. Some of the more advanced tools in use today are “closed loop referral systems (CLRS)”, where not only is the referral (the prescription) captured, but also the status and resolution. For example, instead of providing a person with a list of food banks near their location, a referral is sent to one or more food-related community-based organizations (CBOs) which are also authenticated users of the referral system. Those CBOs may then accept or reject the referral based on their capacity ( *i.e.* , if they have the necessary resources and if the person in need meets their criteria for services, whether by age, veteran status, etc.) and then update the status once the service has been received. North Carolina's NCCARE360 [ 28 ] is an example of a CLRS, highlighted as “Innovation in Action” in the SDOH Playbook [ 20 ].

### 3.1. Clinical Screening Data

Assessment of adverse SDOH is increasingly recognized as an important aspect affecting care delivery and healthcare outcomes [ 29 30 31 32 ]. Unfortunately, these risk factors are not reliably captured in the EHR [ 33 34 35 ]. Where data quality issues are observed, they often further exacerbate demographic disparities, with data missing “not at random” between different racial and ethnic groups [ 36 , 37 ]. While screening for SDOH measures is important, the process can be time-consuming and resource-intensive in healthcare settings, adding complexity to already overloaded clinical encounters [ 38 , 39 ]. To add to the challenge, SDOH domains have not been reliably captured within structured data elements in the EHR or in standardized formats that promote interoperability and digital sharing [ 40 ].

The lack of standardization in how SDOH data is collected in clinical IT systems is evident with the plethora of screening tools administered and the variety of data standards applied to document and exchange this information across disparate systems and networks. There are multiple validated SDOH screening questionnaires for diverse populations, only some of which have been mapped to existing data standards [ 41 42 43 ]. Technology for digital screening includes health portals and tablets, but it is not universally available in clinical care. If it is not available, then more traditional paper screenings or in-person verbal formats are required. EHR vendors may have specific SDOH data capture options but also allow for local configuration, adding to the lack of standardization [ 33 ]. For unstructured SDOH documentation, translation into usable data requires additional processing outside of clinical care using natural language processing (NLP) [ 44 45 46 ].

Direct digital formats for patient specific SDOH screening are preferable for their efficiency in data capture. In the context of a clinical encounter, there are two primary options: health portal questionnaires or tablet-based surveys. Health portal questionnaires can be sent out to the patient before a visit. The use of health portals among at-risk populations is lower than in other groups. Thus, the capture of SDOH data through these portals may be lower [ 47 ]. At the health system in which one co-author is employed, even with more than 75% of patients with a health portal account, completion of the SDOH questionnaire hovers around 30%. With national health portal usage statistics around 60%, this format can only be responsible for a minority of the total collection [ 48 ]. In-person tablet surveys can help in SDOH data collection but require technology resources not available in many clinical care environments.

While many large providers and health plans have adopted the above technologies in their workflow, for many patients the most common format for screening is a paper survey at the time of a clinical encounter. The most significant limitation of this format is that it still requires a healthcare team member to translate responses into the patient's chart. Additionally, when the survey is administered verbally by the health provider, the potential emotional vulnerability and the time it takes to complete (locally 3-5 minutes) make this option the easiest to omit in a time-limited visit [ 49 ].

For patients who have not been screened systematically, unstructured free-text narratives in clinical notes in the EHR are an additional source of SDOH data. This method has the benefit of being more specific to the patient's circumstance but is not accessible discretely without additional NLP processing or labor-intensive manual review [ 33 ]. While there are many examples of research extracting individual-level SDOH domains from clinical notes, most of these examples are limited to a few domains and are not immediately available for clinical care [ 46 , 50 , 51 ]. International Classification of Disease (ICD), Version 10, Clinical Modification (CM) “Z codes,” used in the U.S., offer an additional option to capture adverse SDOH needs but are only used in a small minority of clinical encounters [ 52 , 53 ]. Similar to clinical documentation narratives, this format requires clinician effort and is relatively nonspecific. In some cases, when patient-specific data is not available, or when a population approach is used, the Area Deprivation Index (ADI) or Social Vulnerability Index (SVI) can be used to infer SDOH status [ 54 55 56 ].

The importance of cultural competence. Direct patient screening can pose challenges as screening for social needs can be a sensitive and potentially stigmatizing experience, requiring empathy and sensitivity to collect most effectively. One benefit of technology-mediated surveys is that they can be self-administered. They produce less emotional vulnerability without specific human interaction. If SDOH screening is performed without appropriate sensitivity and cultural competence, the therapeutic relationship may be compromised. Moreover, if screening is performed without ready intervention, additional harm may be done [ 57 ]. To avoid these adverse outcomes, best practices for screening and referral include: use of screening tools that are appropriate in terms of culture, language, and literacy; integration of screening into existing clinical workflows; universal screening; and a solid referral base [ 58 ]. When screening is completed, however it is done, the clinical team needs to recognize the results and intervene on behalf of the patient.

### 3.2. mHealth

Mobile health (mHealth) technologies and applications are shifting how health data is collected and utilized. Traditional health data collection methods, often centered on clinical factors, fail to capture the nonclinical aspects that significantly influence healthcare outcomes fully. In contrast, mHealth technologies can help introduce a broader, more holistic approach to this issue [ 59 ]. These technologies enable real-time monitoring and gathering of health-related data in everyday settings. As a result, this approach can offer a more comprehensive view of patient health beyond clinical environments. By integrating nonclinical factors into health data collection and deepening our understanding of patient health within daily life contexts, we can create healthcare solutions more closely tailored to the distinct health needs of individuals and communities.

Utilizing mHealth data is becoming increasingly important for healthcare professionals, researchers, and patients [ 60 ]. This data can help patients with limited access to clinical services and bridge information gaps during clinical encounters. A particularly significant and promising aspect of mHealth data is its potential to meet the healthcare needs of historically underserved communities [ 61 ]. mHealth apps enable users to track and manage symptoms of less-researched chronic diseases [ 62 ]. For example, an app called Phendo 1 helps facilitate personalized monitoring and generate valuable patient-reported data in the context of endometriosis. The data captured in this app is vital as it enables individuals to comprehend their health conditions better and communicate more effectively with healthcare providers, fostering the development of customized treatment plans [ 62 ].

mHealth data is also instrumental in managing mental health issues, such as anxiety and depression, especially for marginalized groups like Black women. Black women frequently face a higher rate of adverse health outcomes related to these conditions [ 63 , 64 ]. Due to systemic barriers restricting their access to standard healthcare services, Black women are not always able to appropriately address these issues. Culturally appropriate mHealth applications can help address this problem by providing Black women with resources tailored to their specific health needs and preferences, thereby increasing their involvement in managing their mental health effectively [ 63 ]. This shift towards leveraging mHealth data is central to advancing precision prevention. It allows for creating interventions that are more accurate, culturally sensitive, and effective, considering the distinct experiences and needs of various groups. This approach marks a significant step towards making healthcare more equitable and accessible, thus ensuring that all individuals, regardless of background, can access necessary healthcare services.

### 3.3. Social Media

Social media emerges as a valuable medium for capturing real-time health experiences of individuals and communities. This kind of data is vital because it can bridge the gaps in health information that might be missing in EHRs or insufficiently discussed and documented during medical visits. Platforms like X (Twitter), Meta (Facebook), Instagram, TikTok, and others serve as platforms where people frequently discuss their health challenges, experiences, and motivations. Social media provides insights into the lived experiences of individuals, particularly in how HRSNs manifest and influence healthcare access and understanding. Notably, platforms like TikTok offer a rich source of video content where users openly share their experiences to foster awareness and peer support and normalize conversations around chronic illnesses. Such data is instrumental in understanding stigmatized topics and informs the development of online health communities tailored to the needs of adolescents and young adults [ 65 ]. Similarly, platforms like Meta, Instagram, and Tumblr provide multimodal data that sheds light on the health experiences of diverse groups, including fat 2 , LGBTQ+, and disabled individuals, in both clinical and non-clinical settings. The experiences of these communities, especially those with intersecting marginalized identities, can significantly differ from those of cis-heteronormative, straight-sized (people who are not fat [ 66 ]), and non-disabled individuals. For example, social media data can reveal the impacts of issues like fatphobia, particularly in online discussions about anti-fatness among individuals with eating disorders [ 66 ]. These platforms are crucial for building online communities, enhancing health understanding, and developing health literacy among such groups. Thus, utilizing social media data is critical to crafting early interventions and addressing major health concerns, considering community input and the unique experiences shared on these platforms. This approach can lead to more effective, tailored preventive health strategies that acknowledge and address the diverse needs and challenges of various groups within the population.

## 4. Data Standardization and Structure

In the section above, we identify different types of data that may be relevant to a precision prevention approach. For social media and mHealth data, storage and exchange tend to be vendor-based. In some cases, application programming interfaces (APIs) enable data access to social media data [ 67 ]. Researchers have made progress leveraging open mHealth standards for the integration of person-generated data (PGD) into the EHR, but we are still in the early days [ 68 , 69 ]. An area where significant progress has been made toward data standardization is in SDOH data in the clinical context. Central to this work is a massive collaboration known as the HL7 Gravity Project.

### 4.1. The HL7 ^®^ Gravity Project

Over the past decade, growing investments in non-clinical interventions [ 70 , 71 ], coupled with the growing number of national “calls to action” [ 72 73 74 ] for health systems to play a more prominent role in fostering whole-person care, have spurred new policies and programs for collecting and using SDOH data in clinical settings. The ability to link clinical and non-clinical data depends on using agreed-upon terms and data standards across the technologies that collect, use, and exchange this information. However, as described above, this standardization has been lacking.

A series of federal rule-making and related programs, beginning with the Health Information Technology for Economic and Clinical Health (HITECH) Act in 2009 and the 21 ^st^ Century Cures Act in 2016, have propelled standard-based approaches to support patient-level electronic data collection and exchange [ 75 ]. The HITECH programs incentivized the use of a common set of medical terminologies and codes to represent concepts such as race, ethnicity, and preferred language but were limited in requirements on other concepts for social risk assessments, diagnosis, goals, and interventions.

In 2019, following a series of multi-stakeholder discussions with experts in SDOH data from healthcare, community health, government, academia, digital health, and health informatics, the Social Interventions Research and Evaluation Network (SIREN), with funding from the Robert Wood Johnson Foundation and in partnership with EMI Advisors, launched the Gravity Project. The Gravity Project is an open multi-stakeholder public collaborative and HL7 Fast Healthcare Interoperability Resource (FHIR ^®^ ) accelerator of over 3,000 participants focused on developing, testing, and publishing national SDOH data standards for use in digital technologies including EHRs [ 76 ]. The data standards support both structural-level interoperability [ 77 ] and semantic-level interoperability. The structural standards incorporate the HL7 FHIR specification, while the semantic standards are specified by the type of clinical activity the SDOH data concept represents. The coded vocabularies and value sets used to represent the SDOH concepts are enumerated in Table 1 . SDOH-related domains and the respective terminologies designated through the Gravity Project..

**Table 1.** SDOH-related domains and the respective terminologies designated through the Gravity Project.

Use	Standard
Screening/assessment question/answer pairs	LOINC®
Observations	SNOMED CT®
Diagnoses	ICD-10
Goal setting	LOINC®
Interventions	SNOMED CT®
Billing	CPT®/ HCPCS (Current Procedural Terminology / Healthcare Common Procedure Coding System)

The Gravity Project further categorizes SDOH interventions into nine types, as presented in Table 2 . SDOH intervention types as defined through the Gravity Project..

**Table 2.** SDOH intervention types as defined through the Gravity Project.

Gravity Term	Gravity Definition	Example
Adjustment	Adjustment of clinical plan to accommodate social risk	Adjustment of clinical plan to accommodate social risk. SNOMED CT 1269404007
Assistance/Assisting	To give support or aid to; help	Assistance with application for food pantry program. SNOMED CT 467771000124109
Coordination	Process of deliberately organizing activities and sharing information to achieve safer and more effective care aligned with patient preferences	Coordination of resources to address food insecurity. SNOMED CT 1004110005
Counseling	Psychosocial procedure that involves listening, reflecting, etc., to facilitate recognition of course of action/solution	Counseling for food insecurity care plan participation barriers. SNOMED CT 464681000124109
Education	Procedure that is synonymous with those activities such as teaching, demonstration, instruction, explanation, and advice that aim to increase knowledge and skills, change behaviors, assist coping and increase adherence to treatment	Education about Senior Farmers' Market Nutrition Program. SNOMED CT 464341000124108
Evaluation of eligibility (for X)	Process of determining eligibility by evaluating evidence	Evaluation of eligibility for Senior Farmers' Market Nutrition Program. SNOMED CT 464651000124101
Evaluation/ Assessment	Determination of a value, conclusion, or inference by evaluating evidence	Assessment for food insecurity. SNOMED CT 1002224003
Provision	To supply/make available for use	Provision of food voucher. SNOMED CT 464411000124104
Referral	The act of directing someone to a different place or person for information, help, or action	Referral to food prescription program. SNOMED CT 464061000124105

As of early 2024, the Gravity Project has published value sets in the National Library of Medicine (NLM) Value Set Authority Center (VSAC)for 19 SDOH domains [ 78 ], including food insecurity, housing instability, homelessness, and transportation insecurity. Value sets are lists of specific values (terms and their codes) that define clinical concepts derived from standard vocabularies to support effective health information exchange. The Gravity Project defined value sets establish boundaries around what terms are allowed for a documented SDOH condition or what terms serve as a denominator for an SDOH quality measure.

In 2021, the Gravity Project SDOH value sets were incorporated into the Office of the National Coordinator for Health IT (ONC) USCDI Version 2 [ 26 ]. The USCDI is a standardized set of health data classes and constituent data elements for nationwide, interoperable health information exchange. As noted in the ONC 2022 Report to Congress [ 79 ], USCDI is effectively the minimum dataset of a health system. It serves as the floor of what data are available via the health IT systems of most hospitals and doctors' offices and the major consumer health apps that give consumers online access to electronic health information. In addition to the core data set within USCDI, ONC also supports an extensible data set, USCDI+, to meet specific programmatic and/or use cases required by federal and industry partners, particularly in the areas of public health, care quality, cancer, behavioral health, and maternal health [ 80 ].

The first version of the USCDI was adopted as a standard in the ONC Cures Act Final Rule [ 81 ] and thereby made a requirement for certified health IT developers to support in their products. The ONC Cures Act Final Rule also includes policies promoting the use of secure, standard-based APIs that encourage the development of health apps that provide access to and use of data in EHRs to better support person-centered care and patient empowerment. In December 2023, ONC published the Health Data, Technology, and Interoperability: Certification Program Updates, Algorithm Transparency, and Information Sharing (HTI-1) Final Rule [ 82 ], which makes updates to the ONC Health IT Certification Program with new standards. The final rule specifically raises the baseline version of the USCDI from Version 1 to Version 3, thereby making it a requirement for certified health IT developers to capture and exchange the USCDI V3 data elements, including SDOH elements and the Gravity Project's relevant value sets, by January 1, 2026. USCDI, by default, is agnostic to the data model and transport mechanisms adopted to exchange electronic information. USCDI provides a common language and meaning to the information that can be collected and exchanged using common data models like FHIR and OMOP (Observational Medical Outcomes Partnership).

### 4.2. The Need for Data Granularity

The work of the Gravity Project and associated data classes included in the USCDI underscore the importance of specifying agreed-upon standards to represent key concepts for documenting individual social risks and social needs in electronic systems. Although distinct coded terminologies are used to differentiate between a social risk screening activity (documented in a LOINC® code) and a clinical diagnosis (documented as an ICD-10 Z code), these concepts are grouped as value sets published in the NLM VSAC. Most recently, the Gravity Project published a crosswalk of coded concepts for the Accountable Healthcare HRSNs screening tool [ 83 ].

Data standards play a key role in precision prevention. First, they promote the collection of individual-level data to facilitate clinical and administrative decision-making and quality improvement. This highlights a significant cultural shift in healthcare providers incorporating the patient's voice in the information they document and use in care delivery. Second, standards facilitate the sharing of this information electronically across disparate systems of care, social services, and research. We begin to move from a unidirectional clinical approach to a bi-directional and multi-directional electronic exchange. Third, they facilitate payment for social risk and social needs data collection and intervention activities. Ultimately, it is about collecting the data once and being able to reuse it for upstream ( *e.g.* , payment model design) and downstream activities ( *e.g.* , claim reimbursement and resource utilization). For example, payment models can address and evaluate SDOH in various ways, including incorporating screenings and referrals, social risk adjustment, direct social services funding, community convening, and health equity statements. The utility of the Gravity Project standards, then, is clearly not limited to the clinical context in which they are used, but also useful for the broader goal of precision prevention.

## 5. Data Use for Precision Prevention

Once data relevant to precision prevention can be captured, stored, and exchanged, it can be leveraged in various contexts to facilitate addressing those needs, thus contributing to the prevention or reduction of illness.

### 5.1. Clinical Setting

Identifying unmet social needs in a clinical setting can facilitate connecting people to resources that can facilitate prevention, but it does add another layer of complexity [ 21 ]. Giving information about resources does not always translate into the person receiving resources. In addition, it is necessary to use trauma informed practices to meet the patient where they are with a need.

Closed-loop referral systems (CLRS) like Findhelp and Unite Us provide extensive resource directories within their technology platforms to connect people with needs to resources to address those needs.[ 84 ] Using a combination of geolocation and CBOs specific requirements for service, these tools match people to the best options for their needs and provide a mechanism to make a referral to these services. The goal of these platforms is to provide a “no wrong door” closed-loop referral where a social needs referral can not only be made but can be tracked from when it is placed to when the resource is provided and the need addressed.

A systematic review by Pourat *et al.* [ 85 ] concluded that while significant progress has been made in collecting SDOH-related data in EHRs, more effort is needed both to take action based on that data and to evaluate outcomes following intervention. Iott *et al.* [ 86 ] found relatively low rates of engagement with SDOH data collected. Clinical locations with dedicated support staff, such as a population health nurse trained in social resource allocation, have been found to be more successful in placing initial referrals [ 21 ]. Even with placing of referrals, there can still be multiple points of failure to a successful connection [ 87 ].

### 5.2. Clinical and Non-clinical Data Linkages

Linking clinical and non-clinical data can also inform tailored and focused resources to impacted communities and individuals to prevent or reduce illness and improve health. For example, the Environmental Justice Index [ 88 ] combines social risk and environmental exposure data with clinical disease burden for a community. This can inform policies and interventions to lower the burden of exposure and disease in an impacted community. Linking clinical data from EHRs ( *e.g.* , people with diabetes) with non-clinical data ( *e.g.* , food insecurity) can help focus care management activities to people with chronic diseases.

We can combine several sources of non-clinical data to identify communities with a higher burden of risk factors, for example the SVI, to focus resources for particular communities. We can further drive action to higher risk populations by linking data sets. For example, by linking data sets in public programs ( *e.g.* , Medicaid, WIC, SNAP), state agencies can identify who may be eligible for a public assistance program, but not enrolled [ 89 ]. This can inform outreach activities. Further linking to data indicative of food insecurity ( *e.g.* , screening data or data relating to referrals to food resources) can prioritize outreach to the highest risk individuals. In addition, linking clinical and non-clinical data sets can inform deployment of resources. For example, robust and granular data on vaccination status by race, ethnicity, and geography combined with SVI of communities can inform strategic deployment of vaccine resources to populations with lower vaccination rates in communities of higher social vulnerability, and did exactly that in the context of COVID-19 [ 90 ].

### 5.3. Population Level

SDOH data can be used to apply targeted precision prevention to different risk tiers in the population [ 23 ]. At the population level, data can be utilized to identify priorities for population health improvement and track progress in improvement through initiatives such as Healthy People 2030 for the nation [ 91 , 92 ] and through state adaptations, such as Healthy North Carolina 2030 [ 93 ]. These full population measures contain both clinical metrics, for example early prenatal care, and non-clinical metrics such as access to healthy foods. Higher risk populations can be identified by disaggregating full population data to identify disparities in *risk factors* , for example people living in poverty. Pre-natal care services and food programs can then be promoted among high-risk communities. Finally, tailored interventions can be targeted for the segment of the population *experiencing* undesirable outcomes, for example populations with higher rates of infant mortality or communities located within food deserts [ 23 ].

## 6. Data Governance Considerations

Some of the data described above, for example self-administered surveys, mHealth data and social media data, are non-clinical data and are collected directly from the subject, in many cases outside of the clinical settings. This has implications related to data governance, including both data quality and privacy issues. While privacy rules around clinical data are well established under Health Insurance Portability and Accountability Act (HIPAA), significant ambiguity remains regarding the collection and re-use of data that does not originate through a HIPAA “covered entity”, *i.e.* , a healthcare provider or payer.

Though it is commonly recognized that clinical data are far from pristine, non-clinical data introduces a whole new realm of data quality issues. These issues apply to data that are actively self-reported through surveys or apps, passively collected through mobile activity trackers, or some combination of these approaches for social media— user-supplied in the moment and aggregated over time by social media companies as an effective longitudinal record of activities and mindset. These methods introduce multiple potential sources of bias including who is providing the data (often affluent white people who can afford technological gadgets) [ 94 ], when they are doing so (even for passive collection methods, an activity tracker may be removed), how accurately the data may be collected (e.g., sensors that are dependent on skin tone) [ 95 ], and how many impressions a given post might have (depending on the algorithm used by a social media company) [ 96 ].

User perceptions of data privacy and concerns related to confidentiality vary widely, both across nations and individuals [ 97 ]. One major concern is the ability to re-identify data that had previously been considered de-identified [ 97 ]. Another major apprehension concerns the security and privacy of their health-related data, mainly due to ambiguity surrounding the types of data collected and stored and uncertainties about who can access this data [ 98 ]. Interestingly, a small study found that a group of older (>45) users appear more concerned about use of data to improve personalization of ads than they are concerned about misuse of personal health data [ 98 ]. Users of mHealth apps dealing with stigmatization, discrimination, sexually transmitted diseases, and mental illness, among other issues, express heightened security concerns [ 98 ]. The landscape has been further complicated in the post-Dobbs era, with significant concerns regarding law enforcement's use of data from applications tracking menstrual cycles [ 99 , 100 ]. mHealth researchers and developers must prioritize user privacy and confidentiality to address these concerns effectively. Additionally, the legal landscape must catch up to where technology is going. To this end, Galvin & DeMuro and others have proposed the concept of a “healthcare fiduciary” [ 97 ]. Unfortunately, even where laws exist, they are often not followed [ 97 , 100 ].

## 7. Conclusions

Significant progress has been made in the field of informatics for precision prevention. This advancement has been catalyzed by numerous incentives including national policies, payment models, program reforms, and quality reporting. Critical developments include the work from the Gravity Project and open value sets, maturation of the USCDI to include SDOH-related domains, increased use of social media and mHealth among specific communities and populations, and the adoption of innovative closed loop referral systems to address social needs. Still, many challenges and opportunities remain in this space.

Certified health IT developers will need to update their systems to support the USCDI Version 3 data classes and thereby adopt coded data elements for the four SDOH data classes no later than January 2026. Given this directive, public and private entities need to further collaborate on education and outreach activities that promote the collection and use of structured SDOH data and the evaluation of SDOH interventions. Currently, awareness of how to integrate clinical and social care data is nascent across most health systems and community-based organizations. Similarly, the limited use of FHIR-based open APIs by local networks for directed exchange (push) and national networks for query-based exchange creates ongoing barriers for interoperability across clinical and social services systems.

There is limited evidence on what SDOH interventions benefit the right population at the right time. Even though nationally recognized data standards exist to represent SDOH interventions across 19 domains, more work is needed to identify what interventions work best for specific populations and groups by race, ethnicity, and language. Relatedly, current funding models do not support ongoing monitoring of the effectiveness of tailored SDOH interventions by population and group. These challenges point to an opportunity to apply the Learning Health System (LHS) model in the context of precision prevention. LHSs are designed to use data-driven learning processes to continuously and iteratively research and improve clinical practice. They leverage the data collected through clinical care to inform research questions and, ultimately, clinical guidelines, which then generate more clinical data. The LHS life cycle is an ideal format to extend and refine the effectiveness of precision prevention health interventions. By tailoring the needs of specific individuals or communities based on their unique risk factor profiles, targeted population-level prevention strategies provide more specific and tangibly effective resources/interventions.

While increased use of mHealth and social media in this space is promising, particularly for traditionally marginalized populations, there is significant uncertainty around the storage, exchange, and use of data that is derived from non-HIPAA covered entities from both a legal and ethical perspective. These topics must be addressed, with significant input from patient and consumer advocates.

## Notes

Box 1: Axes of distinction for precision prevention and related terms **How many** - all people **→** group **→** individual
**Presence of good vs. absence of bad** - thriving **→** not sick **→** sick
**Level of risk** - entire population **→** at risk individuals **→** those actually experiencing an adverse event or outcome
**Intervention** - 1, 2, 3 prevention **→** treatment
**Actors** - individual **→** clinical care provider **→** social service provider **→** public health practitioner **→** government

## Footnotes

1
https://citizenendo.org/phendo/

2
As explained in [ 66 ], the word “fat” was chosen consciously as a neutral descriptor, rather than use of a medicalized term such as “overweight” or “obese”, which suggest the need for a cure to a naturally occurring difference.

## References

[1] Guttmacher AE, Collins FS. Genomic Medicine — A Primer. N Engl J Med 2002; 347: 1512–1520. https://doi.org/10.1056/NEJMra012240 10.1056/NEJMra012240 12421895

[2] Chan IS, Ginsburg GS. Personalized Medicine: Progress and Promise. Annu Rev Genomics Hum Genet 2011; 12: 217–244. https://doi.org/10.1146/annurev-genom-082410-101446 10.1146/annurev-genom-082410-101446 21721939

[3] Hood L, Flores M. A personal view on systems medicine and the emergence of proactive P4 medicine: predictive, preventive, personalized and participatory. New Biotechnol 2012; 29: 613–624. https://doi.org/10.1016/j.nbt.2012.03.004 10.1016/j.nbt.2012.03.004 22450380

[4] Collins FS, Varmus H. A New Initiative on Precision Medicine. N Engl J Med 2015; 372: 793–795. https://doi.org/10.1056/NEJMp1500523 10.1056/NEJMp1500523 PMC5101938 25635347

[5] The “All of Us” Research Program. N Engl J Med 2019; 381: 668–676. https://doi.org/10.1056/NEJMsr1809937 10.1056/NEJMsr1809937 PMC8291101 31412182

[6] Jaffe S. Planning for US Precision Medicine Initiative underway. The Lancet 2015; 385: 2448–2449. https://doi.org/10.1016/S0140-6736(15)61124-2 10.1016/S0140-6736(15)61124-2 26122056

[7] Khoury MJ. The Success of Precision Medicine Requires a Public Health Perspective Blogs CDC. 2015; Internet: https://blogs.cdc.gov/genomics/2015/01/29/precision-medicine/; Accessed: 07.01.2024

[8] Jefferson T. Preface: Without health there is no happiness. In: The Future of the Public's Health in the 21st Century. National Academies Press (US); 2002; Internet: https://www.ncbi.nlm.nih.gov/books/NBK221223/. Accessed: 29.04.2024

[9] What is Population Health? | Population Health Training | CDC. 2023; Internet: https://archive.cdc.gov/www_cdc_gov/pophealthtraining/whatis.html; Accessed: 25.02.2024

[10] Bharel M, Mohta NS. Defining Distinctions Between Public and Population Health to Knock Down Barriers That Impede Care. Catal Non-Issue Content 2020; 1. https://doi.org/10.1056/CAT.20.0432

[11] Breslow L. From Disease Prevention to Health Promotion. JAMA 1999; 281: 1030–1033. https://doi.org/10.1001/jama.281.11.1030 10.1001/jama.281.11.1030 10086439

[12] Prosperi M, Min JS, Bian J, Modave F. Big data hurdles in precision medicine and precision public health. BMC Med Inform Decis Mak 2018; 18: 139. https://doi.org/10.1186/s12911-018-0719-2 10.1186/s12911-018-0719-2 PMC6311005 30594159

[13] Precision Public Health and Precision Medicine: Two Peas in a Pod | Blogs | CDC. 2015; Internet: https://blogs.cdc.gov/genomics/2015/03/02/precision-public/; Accessed: 25.02.2024

[14] Steele FR. Personalized medicine: something old, something new. Pers Med 2009; 6: 1–5. https://doi.org/10.2217/17410541.6.1.1 10.2217/17410541.6.1.1 29783381

[15] Rebbeck TR. Precision Prevention of Cancer. Cancer Epidemiol Biomarkers Prev 2014; 23: 2713–2715. https://doi.org/10.1158/1055-9965.EPI-14-1058 10.1158/1055-9965.EPI-14-1058 25362191

[16] Gillman MW, Hammond RA. Precision Treatment and Precision Prevention: Integrating “Below and Above the Skin”. JAMA Pediatr 2016; 170: 9–10. https://doi.org/10.1001/jamapediatrics.2015.2786 10.1001/jamapediatrics.2015.2786 PMC4705446 26595371

[17] Pearson TA, Califf RM, Roper R, Engelgau MM, Khoury MJ, Alcantara C, et al. Precision Health Analytics With Predictive Analytics and Implementation Research: JACC State-of-the-Art Review. J Am Coll Cardiol 2020; 76: 306–320. https://doi.org/10.1016/j.jacc.2020.05.043 10.1016/j.jacc.2020.05.043 32674794

[18] County Health Rankings Model | County Health Rankings & Roadmaps. Internet: https://www.countyhealthrankings.org/explore-health-rankings/county-health-rankings-model; Accessed: 29.12.2023

[19] Novilla MLB, Goates MC, Leffler T, Novilla NKB, Wu C-Y, Dall A, et al. Integrating Social Care into Healthcare: A Review on Applying the Social Determinants of Health in Clinical Settings. Int J Environ Res Public Health 2023; 20: 6873. https//doi.org/10.3390/ijerph20196873 10.3390/ijerph20196873 PMC10573056 37835143

[20] SDOH-Playbook-3.pdf [Internet]. [cited 2023 Dec 29]. Available from: https://www.whitehouse.gov/wp-content/uploads/2023/11/SDOH-Playbook-3.pdf

[21] McPeek Hinz E, Avery C, Johnson S, Drake C, Spratt S. Addressing Health-Related Social Needs Through Systematic Screening and Integration of a Social Care Technology Platform. NEJM Catal 2023; 4. https://doi.org/10.1056/CAT.22.0324

[22] SDOH-Action-Plan-At-a-Glance.pdf [Internet] [cited 2024 Apr 13]. Available from: https://aspe.hhs.gov/sites/default/files/documents/aabf48cbd391be21e5186eeae728ccd7/SDOH-Action-Plan-At-a-Glance.pdf

[23] Winston FK, Puzino K, Romer D. Precision prevention: time to move beyond universal interventions. Inj Prev 2016; 22: 87–91. https://doi.org/10.1136/injuryprev-2015-041691 10.1136/injuryprev-2015-041691 26271260

[24] Aagaard-Hansen J, Hindhede AL, Terkildsen Maindal H. A conceptual framework for selecting appropriate populations for public health interventions. Front Public Health 2023; 11: 1161034. https://doi.org/10.3389/fpubh.2023.1161034 10.3389/fpubh.2023.1161034 PMC10197960 37213650

[25] Kang E, Jethani P, Foster ER. Person-centered goal setting is feasible in people with Parkinson's disease who have subjective cognitive decline: a mixed methods study. Disabil Rehabil 2023; 45: 90–97. https://doi.org/10.1080/09638288.2022.2025930 10.1080/09638288.2022.2025930 PMC9719695 35023794

[26] United States Core Data for Interoperability (USCDI). . Internet: http://www.healthit.gov/isa/united-states-core-data-interoperability-uscdi; Accessed: 30.12.2023

[27] Morris MA, Yee S, Breslin ML, Savage M, Swenor BK. Health Care Equity Requires Standardized Disability Data In The EHR. Health Aff Forefr 2022. https://doi.org/10.1377/forefront.20221026.83825

[28] NCCARE360 | NCDHHS. Internet: https://www.ncdhhs.gov/about/department-initiatives/healthy-opportunities/nccare360; Accessed: 31.12.2023

[29] Kepper MM, Walsh-Bailey C, Prusaczyk B, Zhao M, Herrick C, Foraker R. The adoption of social determinants of health documentation in clinical settings. Health Serv Res 2023; 58: 67–77. https://doi.org/10.1111/1475-6773.14039 10.1111/1475-6773.14039 PMC9836948 35862115

[30] Hacker K, Auerbach J, Ikeda R, Philip C, Houry D. Social Determinants of Health-An Approach Taken at CDC. J Public Health Manag Pract 2022; 28: 589–594. https://doi.org/10.1097/PHH.0000000000001626 10.1097/PHH.0000000000001626 PMC9555578 36194813

[31] De Lew N, Sommers BD. Addressing Social Determinants of Health in Federal Programs. JAMA Health Forum 2022; 3: e221064. https://doi.org/10.1001/jamahealthforum.2022.1064 10.1001/jamahealthforum.2022.1064 36218886

[32] Bakken S, Dreisbach C. Informatics and data science perspective on Future of Nursing 2020–2030: Charting a pathway to health equity. Nurs Outlook 2022; 70: S77–S87. https://doi.org/10.1016/j.outlook.2022.04.004 10.1016/j.outlook.2022.04.004 36446542

[33] Wang M, Pantell MS, Gottlieb LM, Adler-Milstein J. Documentation and review of social determinants of health data in the EHR: measures and associated insights. J Am Med Inform Assoc 2021; 28: 2608–2616. https://doi.org/10.1093/jamia/ocab194 10.1093/jamia/ocab194 PMC8633631 34549294

[34] Freij M, Dullabh P, Lewis S, Smith SR, Hovey L, Dhopeshwarkar R. Incorporating Social Determinants of Health in Electronic Health Records: Qualitative Study of Current Practices Among Top Vendors. JMIR Med Inform 2019; 7: e13849. https://doi.org/10.2196/13849 10.2196/13849 PMC6592390 31199345

[35] Trochez RJ, Sharma S, Stolldorf DP, Mixon AS, Novak LL, Rajmane A, et al. Screening Health-Related Social Needs in Hospitals: A Systematic Review of Health Care Professional and Patient Perspectives. Popul Health Manag 2023; 26: 157–167. https://doi.org/10.1089/pop.2022.0279 10.1089/pop.2022.0279 PMC10278007 37092962

[36] Cook LA, Sachs J, Weiskopf NG. The quality of social determinants data in the electronic health record: a systematic review. J Am Med Inform Assoc 2021; 29: 187–196. https://doi.org/10.1093/jamia/ocab199 10.1093/jamia/ocab199 PMC8714289 34664641

[37] Berg K, Doktorchik C, Quan H, Saini V. Automating data collection methods in electronic health record systems: a Social Determinant of Health (SDOH) viewpoint. Health Syst 2023; 12: 472–480. https://doi.org/10.1080/20476965.2022.2075796 10.1080/20476965.2022.2075796 PMC10791104 38235302

[38] Yan AF, Chen Z, Wang Y, Campbell JA, Xue Q-L, Williams MY, et al. Effectiveness of Social Needs Screening and Interventions in Clinical Settings on Utilization, Cost, and Clinical Outcomes: A Systematic Review. Health Equity 2022; 6: 454–475. https://doi.org/10.1089/heq.2022.0010 10.1089/heq.2022.0010 PMC9257553 35801145

[39] Lindenfeld Z, Chen K, Kapur S, Chang JE. Assessing Differences in Social Determinants of Health Screening Rates in a Large, Urban Safety-Net Health System. J Prim Care Community Health 2023; 14. https://doi.org/10.1177/21501319231207713 10.1177/21501319231207713 PMC10624082 37916515

[40] Phuong J, Hong S, Palchuk MB, Espinoza J, Meeker D, Dorr DA, et al. Advancing Interoperability of Patient-level Social Determinants of Health Data to Support COVID-19 Research. AMIA Jt Summits Transl Sci Proc 2022: 396–405 PMC9285174 35854720

[41] Moen M, Storr C, German D, Friedmann E, Johantgen M. A Review of Tools to Screen for Social Determinants of Health in the United States: A Practice Brief. Popul Health Manag 2020; 23: 422–429. https://doi.org/10.1089/pop.2019.0158 10.1089/pop.2019.0158 PMC7864106 31910355

[42] Chung EK, Siegel BS, Garg A, Conroy K, Gross RS, Long DA, et al. Screening for Social Determinants of Health Among Children and Families Living in Poverty: A Guide for Clinicians. Curr Probl Pediatr Adolesc Health Care 2016; 46: 135–153. https://doi.org/10.1016/j.cppeds.2016.02.004 10.1016/j.cppeds.2016.02.004 PMC6039226 27101890

[43] Sokol R, Austin A, Chandler C, Byrum E, Bousquette J, Lancaster C, et al. Screening Children for Social Determinants of Health: A Systematic Review. Pediatrics 2019; 144: e20191622. https://doi.org/10.1542/peds.2019-1622 10.1542/peds.2019-1622 PMC6996928 31548335

[44] Lybarger K, Dobbins NJ, Long R, Singh A, Wedgeworth P, Uzuner Ö, et al. Leveraging natural language processing to augment structured social determinants of health data in the electronic health record. J Am Med Inform Assoc 2023; 30: 1389–1397. https://doi.org/10.1093/jamia/ocad073 10.1093/jamia/ocad073 PMC10354760 37130345

[45] Han S, Zhang RF, Shi L, Richie R, Liu H, Tseng A, et al. Classifying social determinants of health from unstructured electronic health records using deep learning-based natural language processing. J Biomed Inform 2022; 127: 103984. https://doi.org/10.1016/j.jbi.2021.103984 10.1016/j.jbi.2021.103984 35007754

[46] Patra BG, Sharma MM, Vekaria V, Adekkanattu P, Patterson OV, Glicksberg B, et al. Extracting social determinants of health from electronic health records using natural language processing: a systematic review. J Am Med Inform Assoc 2021; 28: 2716–2727. https://doi.org/10.1093/jamia/ocab170 10.1093/jamia/ocab170 PMC8633615 34613399

[47] Casillas A, Abhat A, Mahajan A, Moreno G, Brown AF, Simmons S, et al. Portals of Change: How Patient Portals Will Ultimately Work for Safety Net Populations. J Med Internet Res 2020; 22: e16835. https://doi.org/10.2196/16835 10.2196/16835 PMC7647808 33094732

[48] Strawley C, Richwine C. Individuals' Access and Use of Patient Portals and Smartphone Health Apps, 2022. 2023 39413226

[49] Schickedanz A, Hamity C, Rogers A, Sharp AL, Jackson A. Clinician Experiences and Attitudes Regarding Screening for Social Determinants of Health in a Large Integrated Health System. Med Care 2019; 57 Suppl 6 Suppl 2: S197–S201. https://doi.org/10.1097/MLR.0000000000001051 10.1097/MLR.0000000000001051 PMC6721844 31095061

[50] Feller DJ, Bear Don't Walk Iv OJ, Zucker J, Yin MT, Gordon P, Elhadad N. Detecting Social and Behavioral Determinants of Health with Structured and Free-Text Clinical Data. Appl Clin Inform 2020; 11: 172–181. https://doi.org/10.1055/s-0040-1702214 10.1055/s-0040-1702214 PMC7056402 32131117

[51] Stemerman R, Arguello J, Brice J, Krishnamurthy A, Houston M, Kitzmiller R. Identification of social determinants of health using multi-label classification of electronic health record clinical notes. JAMIA Open 2021; 4: ooaa069. https://doi.org/10.1093/jamiaopen/ooaa069 10.1093/jamiaopen/ooaa069 PMC8423426 34514351

[52] Truong HP, Luke AA, Hammond G, Wadhera RK, Reidhead M, Joynt Maddox KE. Utilization of Social Determinants of Health ICD-10 Z-Codes Among Hospitalized Patients in the United States, 2016-2017. Med Care 2020; 58: 1037–1043. https://doi.org/10.1097/MLR.0000000000001418 10.1097/MLR.0000000000001418 PMC7666017 32925453

[53] Guo Y, Chen Z, Xu K, George TJ, Wu Y, Hogan W, et al. International Classification of Diseases, Tenth Revision, Clinical Modification social determinants of health codes are poorly used in electronic health records. Medicine (Baltimore) 2020; 99: e23818. https://doi.org/10.1097/MD.0000000000023818 10.1097/MD.0000000000023818 PMC7769291 33350768

[54] Lines LM, Long MC, Zangeneh S, DePriest K, Piontak J, Humphrey J, et al. Composite Indices of Social Determinants of Health: Overview, Measurement Gaps, and Research Priorities for Health Equity. Popul Health Manag 2023; 26: 332–340. https://doi.org/10.1089/pop.2023.0106 10.1089/pop.2023.0106 37824819

[55] Rollings KA, Noppert GA, Griggs JJ, Melendez RA, Clarke PJ. Comparison of two area-level socioeconomic deprivation indices: Implications for public health research, practice, and policy. PloS One 2023; 18: e0292281. https://doi.org/10.1371/journal.pone.0292281 10.1371/journal.pone.0292281 PMC10553799 37797080

[56] Jain V, Al Rifai M, Khan SU, Kalra A, Rodriguez F, Samad Z, et al. Association Between Social Vulnerability Index and Cardiovascular Disease: A Behavioral Risk Factor Surveillance System Study. J Am Heart Assoc 2022; 11: e024414. https://doi.org/10.1161/JAHA.121.024414 10.1161/JAHA.121.024414 PMC9375494 35904206

[57] Johnson CB, Luther B, Wallace AS, Kulesa MG. Social Determinants of Health: What Are They and How Do We Screen. Orthop Nurs 2022; 41: 88. https://doi.org/10.1097/NOR.0000000000000829 10.1097/NOR.0000000000000829 35358126

[58] Berry C, Paul M, Massar R, Marcello RK, Krauskopf M. Social Needs Screening and Referral Program at a Large US Public Hospital System, 2017. Am J Public Health 2020; 110: S211–S214. https://doi.org/10.2105/AJPH.2020.305642 10.2105/AJPH.2020.305642 PMC7362691 32663088

[59] Rogers CC, Jang SS, Tidwell W, Shaughnessy S, Milburn J, Hauck FR, et al. Designing mobile health to align with the social determinants of health. Front Digit Health 2023; 5: 1193920. https://doi.org/10.3389/fdgth.2023.1193920 10.3389/fdgth.2023.1193920 PMC10232872 37274765

[60] Dunn J, Runge R, Snyder M. Wearables and the medical revolution. Pers Med 2018; 15: 429–448. https://doi.org/10.2217/pme-2018-0044 10.2217/pme-2018-0044 PMC12294383 30259801

[61] Brewer LC, Fortuna KL, Jones C, Walker R, Hayes SN, Patten CA, et al. Back to the Future: Achieving Health Equity Through Health Informatics and Digital Health. JMIR MHealth UHealth 2020; 8: e14512. https://doi.org/10.2196/14512 10.2196/14512 PMC6996775 31934874

[62] Pichon A, Schiffer K, Horan E, Massey B, Bakken S, Mamykina L, et al. Divided We Stand: The Collaborative Work of Patients and Providers in an Enigmatic Chronic Disease. Proc ACM Hum-Comput Interact 2021; 4: 261. https://doi.org/10.1145/3434170 10.1145/3434170 PMC8112593 33981961

[63] McCall T, Threats M, Pillai M, Lakdawala A, Bolton CS. Recommendations for design of a mobile application to support management of anxiety and depression among Black American women. Front Digit Health 2022; 4: 1028408. https://doi.org/10.3389/fdgth.2022.1028408 10.3389/fdgth.2022.1028408 PMC9816326 36620185

[64] Vance MM, Wade JM, Brandy M, Webster AR. Contextualizing Black Women's Mental Health in the Twenty-First Century: Gendered Racism and Suicide-Related Behavior. J Racial Ethn Health Disparities 2023; 10: 83–92. https://doi.org/10.1007/s40615-021-01198-y 10.1007/s40615-021-01198-y 34984654

[65] Zehrung R, Chen Y. Self-Expression and Sharing around Chronic Illness on TikTok. AMIA Annu Symp Proc. 2023; 1334–1343 PMC10785898 38222376

[66] Payne BH, Taylor J, Spiel K, Fiesler C. How to Ethically Engage Fat People in HCI Research. In: Computer Supported Cooperative Work and Social Computing. Minneapolis MN USA: ACM; 2023: 117–121. https://doi.org/10.1145/3584931.3606987

[67] Acker A, Kreisberg A. Social media data archives in an API-driven world. Arch Sci 2020; 20: 105–123. https//doi.org/10.1007/s10502-019-09325-9

[68] Zeng B, Bove R, Carini S, Lee JS-J, Pollak JP, Schleimer E, et al. Standardized Integration of Person-Generated Data Into Routine Clinical Care. JMIR MHealth UHealth 2022; 10: e31048. https://doi.org/10.2196/31048 10.2196/31048 PMC8874926 35142627

[69] Falkenhein I, Bernhardt B, Gradwohl S, Brandl M, Hussein R, Hanke S. Wearable Device Health Data Mapping to Open mHealth and FHIR Data Formats. Stud Health Technol Inform 2023; 305: 341–344. https://doi.org/10.3233/SHTI230500 10.3233/SHTI230500 37387034

[70] Published: Medicaid Waiver Tracker: Approved and Pending Section 1115 Waivers by State. KFF 2023; Internet: https://www.kff.org/medicaid/issue-brief/medicaid-waiver-tracker-approved-and-pending-section-1115-waivers-by-state/; Accessed: 30.12.2023

[71] Thompson FJ, Farnham J, Tiderington E, Gusmano MK, Cantor JC. Medicaid Waivers and Tenancy Supports for Individuals Experiencing Homelessness: Implementation Challenges in Four States. Milbank Q 2021; 99: 648–692. https://doi.org/10.1111/1468-0009.12514 10.1111/1468-0009.12514 PMC8452367 33904611

[72] Integrating Social Care into the Delivery of Health Care: Moving Upstream to Improve the Nation's Health. Washington, D.C.: National Academies Press; 2019 31940159

[73] NASDOH1.pdf [Internet] cited 2024 Apr 13. Available from: https://nasdoh.org/wp-content/uploads/2023/01/NASDOH1.pdf

[74] NQF: NQF and the Aetna Foundation Map Approach to Overcome the Health Impact of Social Determinants. Internet: https://www.qualityforum.org/Overcoming_the_Health_Impact_of_Social_Determinants.aspx; Accessed: 30.12.2023

[75] Health IT Legislation | HealthIT.gov. Internet: https://www.healthit.gov/topic/laws-regulation-and-policy/health-it-legislation; Accessed: 30.12.2023

[76] Gravity Project. Internet: https://thegravityproject.net/; Accessed: 30.12.2023

[77] Interoperability in Healthcare | HIMSS. Internet: https://www.himss.org/resources/interoperability-healthcare; Accessed: 30.12.2023

[78] Social Risk Domain Build - Gravity Project - Confluence. Internet: https://confluence.hl7.org/display/GRAV/Social+Risk+Domain+Build; Accessed: 30.12.2023

[79] HHS ONC. 2022 Report to Congress: Update on the Access, Exchange, and Use of Electronic Health Information. Internet: https://www.healthit.gov/sites/default/files/page/2023-02/2022_ONC_Report_to_Congress.pdf; Accessed: 29.04.2024

[80] USCDI+ | HealthIT.gov. Internet: https://www.healthit.gov/topic/interoperability/uscdi-plus; Accessed: 14.01.2024

[81] HHS ONC. The ONC Cures Act Final Rule. Internet: https://www.healthit.gov/sites/default/files/page2/2020-03/TheONCCuresActFinalRule.pdf; Accessed: 29.04.2024

[82] Health Data, Technology, and Interoperability: Certification Program Updates, Algorithm Transparency, and Information Sharing (HTI-1) Final Rule | HealthIT.gov. Internet: https://www.healthit.gov/topic/laws-regulation-and-policy/health-data-technology-and-interoperability-certification-program; Accessed: 30.12.2023

[83] Resources for Social Risk Coding in Care Settings - Gravity Project - Confluence. Internet: https://confluence.hl7.org/display/GRAV/Resources+for+Social+Risk+Coding+in+Care+Settings; Accessed: 30.12.2023

[84] Cartier Y, Fichtenberg C, Gottlieb LM. Implementing Community Resource Referral Technology: Facilitators And Barriers Described By Early Adopters. Health Aff Proj Hope 2020; 39: 662–669. https://doi.org/10.1377/hlthaff.2019.01588 10.1377/hlthaff.2019.01588 32250665

[85] Pourat N, Lu C, Huerta DM, Hair BY, Hoang H, Sripipatana A. A Systematic Literature Review of Health Center Efforts to Address Social Determinants of Health. Med Care Res Rev 2023; 80: 255–265. https://doi.org/10.1177/10775587221088273 10.1177/10775587221088273 35465766

[86] Iott BE, Adler-Milstein J, Gottlieb LM, Pantell MS. Characterizing the relative frequency of clinician engagement with structured social determinants of health data. J Am Med Inform Assoc JAMIA 2023; 30: 503–510. https://doi.org/10.1093/jamia/ocac251 10.1093/jamia/ocac251 PMC9933071 36545752

[87] Schweitzer A, Mohta NS. Pathways to Success in Meeting Health-Related Social Needs. NEJM Catal 2023; 4: CAT.22.0352. https://doi.org/10.1056/CAT.22.0352

[88] CDC. Environmental Justice Index (EJI). Cent Dis Control Prev 2023; Internet: https://www.atsdr.cdc.gov/placeandhealth/eji/index.html; Accessed: 30.12.2023

[89] Carolina N. Medicaid and SNAP Data Coordination Case Studies. Internet: https://www.chcs.org/media/Medicaid-and-SNAP-Data-Coordination-Case-Studies_South-Dakota_March-2023.pdf; Accessed: 29.04.2023

[90] Peebles A. One U.S. State's Laser Focus on Data Helps Shrink Racial Vaccine Gap. Bloomberg.com 2021

[91] McGinnis JM. Healthy People 2030: A Compass in the Storm. J Public Health Manag Pract 2021; 27: S213–S214. https://doi.org/10.1097/PHH.0000000000001328 10.1097/PHH.0000000000001328 PMC8478301 33797502

[92] National Academies of Sciences, Engineering, and Medicine; Health and Medicine Division; Board on Population Health and Public Health Practice; Committee on Informing the Selection of Leading Health Indicators for Healthy People 2030. Criteria for Selecting the Leading Health Indicators for Healthy People 2030. Washington (DC): National Academies Press (US); 2019. https://doi.org/10.17226/25531 31940161

[93] Thomas AB, Dail K. Creating a Healthy North Carolina: Developing and Reaching Goals for the 21st Century. N C Med J 2021; 82: 179–183. https://doi.org/10.18043/ncm.82.3.179 10.18043/ncm.82.3.179 33972275

[94] Yfantidou S, Sermpezis P, Vakali A, Baeza-Yates R. Uncovering Bias in Personal Informatics. Proc ACM Interact Mob Wearable Ubiquitous Technol 2023; 7: 1–30. https://doi.org/10.1145/3610914

[95] Bent B, Goldstein BA, Kibbe WA, Dunn JP. Investigating sources of inaccuracy in wearable optical heart rate sensors. Npj Digit Med 2020; 3: 1–9. https://doi.org/10.1038/s41746-020-0226-6 10.1038/s41746-020-0226-6 PMC7010823 32047863

[96] Flam F. Musk Promised Transparency, Then Hid Twitter Data. Bloomberg.com 2023

[97] Galvin HK, DeMuro PR. Developments in Privacy and Data Ownership in Mobile Health Technologies, 2016-2019. Yearb Med Inform 2020; 29: 32–43. https://doi.org/10.1055/s-0040-1701987 10.1055/s-0040-1701987 PMC7442507 32823298

[98] Schroeder T, Haug M, Gewald H. Data Privacy Concerns Using mHealth Apps and Smart Speakers: Comparative Interview Study Among Mature Adults. JMIR Form Res 2022; 6: e28025. https://doi.org/10.2196/28025 10.2196/28025 PMC9237761 35699993

[99] Salvatore GM, Bercovitz I, Arigo D. Women's comfort with mobile applications for menstrual cycle self-monitoring following the overturning of Roe v. Wade. mHealth 2023; 10: 1. https://doi.org/10.21037/mhealth-23-31 10.21037/mhealth-23-31 PMC10839505 38323149

[100] Alfawzan N, Christen M, Spitale G, Biller-Andorno N. Privacy, Data Sharing, and Data Security Policies of Women's mHealth Apps: Scoping Review and Content Analysis. JMIR MHealth UHealth 2022; 10: e33735. https://doi.org/10.2196/33735 10.2196/33735 PMC9123546 35522465

